# Key Parameters on the Microwave Assisted Synthesis of Magnetic Nanoparticles for MRI Contrast Agents

**DOI:** 10.1155/2017/8902424

**Published:** 2017-12-04

**Authors:** Maria Eugênia Fortes Brollo, Sabino Veintemillas-Verdaguer, Cesar Menor Salván, Maria del Puerto Morales

**Affiliations:** ^1^Institute of Material Science of Madrid, ICMM-CSIC, Sor Juana Inés de la Cruz 3, 28049 Madrid, Spain; ^2^School of Chemistry and Biochemistry, Georgia Institute of Technology, 315 Ferst Drive NW, Atlanta, GA 30332, USA; ^3^IMIDRA, Finca El Encin, Autovía del Noreste A-2, Km. 38.200, Alcalá de Henares, 28805 Madrid, Spain

## Abstract

Uniform iron oxide magnetic nanoparticles have been synthesized using a microwave assisted synthesis method in organic media and their colloidal, magnetic, and relaxometric properties have been analyzed after its transference to water and compared with those nanoparticles prepared by thermal decomposition in organic media. The novelty of this synthesis relies on the use of a solid iron oleate as precursor, which assures the reproducibility and scalability of the synthesis, and the microwave heating that resulted in being faster and more efficient than traditional heating methods, and therefore it has a great potential for nanoparticle industrial production. The effect of different experimental conditions such as the solvent, precursor, and surfactant concentration and reaction time as well as the transference to water is analyzed and optimized to obtain magnetic iron oxide nanoparticles with sizes between 8 and 15 nm and finally colloids suitable for their use as contrast agents on Magnetic Resonance Imaging (MRI). The *r*_2_ relaxivity values normalized to the square of the saturation magnetization were shown to be constant and independent of the particle size, which means that the saturation magnetization is the main parameter controlling the efficiency of these magnetic nanoparticles as MRI *T*_2_-contrast agents.

## 1. Introduction

In the last decade a new synthesis approach of nanoparticles based on the use of microwave dielectric heating has gained a lot of attention because of its versatility in different application areas, such as polymer chemistry, biomedicine, material science, and nanotechnology [1]. This nonclassical heating has shown an impressive reduction in synthesis time, from hours to minutes, increased product yield, and improved material properties, when compared to the conventional heating (by convective heat transfer), improving its reproducibility [1–3]. This synthesis approach seems to be especially interesting for the synthesis of nanoparticles for biomedical applications such as magnetic iron oxide [4], particularly in relation to the transference of the technology to the clinic and the need for standardization to avoid batch to batch inhomogeneity [5].

The traditional heating, transferring energy from the reaction vessel to the reactant mixture by forced convection depends on viscosity and thermal conductivity of the fluid to be heated; temperature gradients are unavoidable in such a system. In contrast, microwave irradiation triggers heating of the overall system by two mechanisms: ionic conduction and dipolar polarization. The charged particles in the mixture contribute with the first one while the dipoles (like a polar solvent) contribute with the second one. The heating is produced by direct coupling of the energy from the microwave with the molecules in the mixture. Thus, the more polar a reaction mixture is, the greater its ability to couple with the microwave energy will be [6] so either the substrate or reagents have to be polar in order to allow sufficient heating by microwaves.

Up to now, microwave synthesis of iron oxide nanoparticles has been carried out mainly for the preparation of ultrasmall nanoparticles in water or alcohol resulting in extremely interesting positive contrast agents for diagnosis [7–10] in Magnetic Resonance Imaging (MRI). On the contrary, uniform flower-like Fe_3_O_4_ clusters of a few *μ*m were fabricated in ethylene glycol with FeCl_3_, sodium acetate and a surfactant, under microwave irradiation for 15 to 60 minutes [11]. An organic solvent like benzyl ether has also been used with microwave heating, but, in that case, addition of a small proportion of ionic liquid was required to obtain magnetite nanoparticles up to 10 nm [12]. The control of the nanoparticle size by this route deserves further investigation in order to use microwave heating for the preparation of nanoparticles not only as *T*_1_ but also as *T*_2_ MRI contrast agents.

With the aim to produce a *T*_2_ MRI contrast agent, iron oxide nanoparticles were prepared in organic media using a microwave assisted synthesis method; then their colloidal and magnetic properties are explored before and after its transference to water and compared with those nanoparticles prepared by thermal decomposition in organic media. The effect of different experimental conditions such as the solvent, Fe concentration, oleic acid/Fe ratio, the heating ramp, and the reaction time is analyzed. For standardization purpose, a unique precursor that is a solid iron oleate, easy to prepare in large quantities, modified from Patent US number 20130089740 [13], easy to handle, and stable for long storage times was used for the first time and properly characterized in comparison to the liquid oleate, traditionally used in the thermal decomposition process [14]. Finally, the colloids are evaluated for MRI imaging measuring *r*_1_ and *r*_2_ relaxivities. The results were related to the particle and hydrodynamic size and magnetic moment per particle using the universal scaling law to predict the efficiency of magnetic nanoparticles as MRI *T*_2_-contrast agents recently reported [15].

## 2. Materials and Methods

### 2.1. Solid Oleate Synthesis

The sodium oleate was synthesized by adding sodium hydroxide (5.91 g) and oleic acid 90% (43.6 g) to 140 ml of hexane and heating the mixture up to 60°C in an oil bath, with magnetic stirring at 400 rpm. After one day at 60°C, a white precipitate of sodium oleate that is dissolved by adding 80 ml of ethanol at the same temperature appears. Then 10.8 g of FeCl_3_ in 80 ml of distilled water is added and the solution boils violently at 57°C. The system is heated for 2 hours more, and then it is chilled with a cold water bath. The denser aqueous phase was eliminated by decantation using a separating funnel; the upper organic phase was filtered with filter paper prior to the precipitation of the solid iron oleate by the addition of an equal volume of methanol. The orange solid iron oleate was redissolved in hexane and reprecipitated with methanol three times. Finally it was dried over P_2_O_5_, milled gently, and stored at room temperature in a desiccator over silica gel. For comparison, a liquid oleate was prepared following a methodology previously reported [14, 16, 17].

### 2.2. Microwave Synthesis

The synthesis of magnetic nanoparticles was carried out using a microwave oven Monowave 300®. This instrument has built-in magnetic stirrer, temperature control by internal fiber-optics probe surface temperature by infrared sensor, and pressure measurement produced by Anton Paar GmbH, Austria, working on 2.45 GHz. Different parameters were explored in the synthesis of oleic acid coated iron oxide nanoparticles by microwave heating such as the nature of the solvents with different dielectric constant (octadecene, dibenzyl ether, benzyl alcohol, phenyl ether, and dimethyl sulfoxide (DMSO)), the Fe concentration, and the heating ramps (2–4°C/min). The reaction mechanism was also explored fixing all the experimental conditions and varying the reaction time from 1/2 h up to 4 hours. Finally, two microwave samples were prepared under selected conditions as follows: a mixture containing 0.15 g of solid iron oleate, 0.76 g of oleic acid, and 8.32 ml of dibenzyl ether (MwE8) or benzyl alcohol (MwA8) was stirred at 600 rpm, while the temperature increases at 3.75°C/min until 250°C and then was maintained at this temperature for 1 hour. For comparison, two samples were prepared by standard thermal decomposition [14, 18] under similar conditions of Fe concentration, oleic acid content, and temperature ramp. In brief, a mixture containing 0.9 g of solid iron oleate, 4.5 g of oleic acid, and 50 ml of dibenzyl ether (TdE12) or octadecene (TdO15) was added on a three-neck round-bottom flask mounted on a temperature-controlled N_2_ reflux system, overhead stirred at 100 rpm until reach 100°C. The temperature was increased in a controlled way, with a heating ramp of 3.75°C/min until reflux temperature, given by boiling point of the solvent, 290°C or 320°C, and this temperature was maintained for 1 hour.

### 2.3. Nanoparticle Coating

Nanoparticles were transferred to an aqueous medium by exchanging the oleic acid at the surface by dimercaptosuccinic acid (DMSA) [19]. For that purpose a solution of 20 ml of toluene containing 50 mg of carefully washed nanoparticles was added to a solution of 90 mg of DMSA in 5 ml of DMSO. The resulting suspension was then gently stirred by rotation for at least 2 days, until 2 phases appear. The resulting nanoparticles were washed with ethanol and centrifuged at 7500 rcf, at least 3 times. The final black solid was air dried and redispersed in distilled water. Diluted sodium hydroxide was added to increase the pH up to 10. The dispersion was then placed in a cellulose membrane tube molecular weight cut-off (MWCO) 10000 Da and dialyzed for 3 days in front of distilled water, to remove any excess of unreacted DMSA. Finally the pH of the dispersion was adjusted to 7 and the dispersion filtered through a polyethylene oxide filter with a pore size of 0.22 *μ*m in order to check its capability for being sterilized by this procedure.

### 2.4. Characterization

The core size and shape of nanoparticles were measured by transmission electron microscopy, where a drop of toluene, in the case of the oleate precursor, or water, in the case of the nanoparticles, was placed on a carbon coated copper grid, allowing all the solvent to evaporate at room temperature. The images were captured at a 100 keV JEOL-JEM 1010 microscope, equipped with a digital camera Gatan model Orius 200 SC, at the Universidad Autónoma de Madrid. Size and size distributions were obtained with the open source software ImageJ, using TEM images and counting at least 300 nanoparticles [ISO13322-1]. A log-normal fit was performed to obtain mean sizes and deviation in number (TEM diameter in number *d* = ∑*x* *dN*/∑*dN*), which can be transformed to a volume distribution in order to compare the values with XRD mean size (TEM diameter in volume = ∑*x*^4^*dN*/∑*x*^3^*dN*, where *x* = particle size and *N* = number of particles) [20].

The iron oxide phase was determined by X-ray diffraction on a Powder Diffractometer Bruker D8 Advance with Cu K*α* radiation with energy-discriminator, in 2*θ* ranging from 10 to 90 degrees, with acquisition time of 5 seconds using 0.05-degree step. Crystal sizes were calculated by the width of the peak with the greatest intensity (311), using the Scherrer equation [21]. Fourier transform infrared spectroscopy (FTIR) spectra were recorded using a Bruker IFS 66VS to confirm the iron oxide phase, the presence, and nature of the coating and its surface bonding. IR spectra were recorded between 4000 and 250 cm^−1^ and the samples were prepared by diluting 2% wt iron oxide powder in KBr and pressing it into a pellet. Quantification of the coating was carried out by simultaneous thermogravimetric (TGA) and differential thermal analysis (DTA) of the samples on a Seiko Exstar 6300 instrument. Samples were heated from room temperature to 900°C at 10°C/min under an air flow of 100 ml/min.

Magnetic characterization was performed on dried powder samples after transference to water using a vibrating sample magnetometer (VSM; MLVSM9 MagLab 9 T, Oxford Instrument). Magnetization temperature dependence was recorded following a ZFC-FC standard protocol: ZFC curve, the sample is cooled down from 290 K to 5 K without any applied magnetic field and then, a small DC magnetic field is applied and the magnetization is recorded as temperature increases up to 290 K; FC curve, the sample is cooled down to 5 K under an applied magnetic field and the magnetization is recorded as temperature increases up to 290 K. To obtain the hysteresis loops, the samples were first demagnetized at fixed temperature and DC magnetization was measured in discrete constant fields during the field sweep. The initial susceptibility (*χ*) was measured in the field range ±100 Oe and the saturation magnetization (*Ms*) was achieved by fitting the magnetization curves at room temperature to the Langevin function.

The hydrodynamic size of the nanoparticle aqueous suspensions at pH 7 was measured by dynamic light scattering (DLS) in a standard cuvette, using a Zetasizer NanoZS device (Malvern Instruments). A laser emitting red light is the energy source with an angle of 173° between the sample and detector. The hydrodynamic size of the particles was measured by photon correlation spectroscopy and expressed in terms of intensity, which reflects better the quality of the sample and number [22]. Zeta Potential was measured as a function of the pH, at room temperature, using KNO_3_ 0.01 M as the electrolyte and HNO_3_ and KOH to adjust the pH.

Finally, MRI relaxometric properties were investigated by measuring the longitudinal (*T*_1_) (sequence t1_ir_mb) and transversal (*T*_2_) (sequence t2_ir_mb) protons relaxation times at different dilutions between 0 and 0.07 mM of Fe in a MINISPEC MQ60 (Bruker) at 37°C and a magnetic field of 1.5 T. The sequences used are original from Bruker.

## 3. Results and Discussion

**Figure 1:**
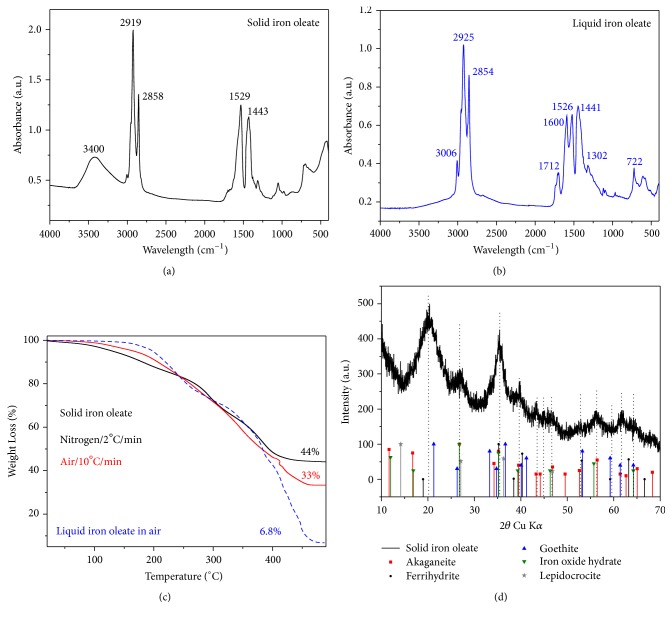
Infrared spectroscopy absorbance of solid (a) and liquid oleate (b) [14, 28]; (c) Thermogravimetric analysis of the Fe oleates (solid and liquid) under different conditions; (d) X-ray diffraction pattern for solid iron oleate.

The microwave assisted synthesis of magnetic nanoparticles in organic media was carried out starting from a solid oleate-Fe precursor, being one of the achievements of this work, the precursor itself. The advantages of having a solid precursor in comparison to a liquid oleate are numerous: first of all, its reproducibility, scalability, easy purification by precipitation, high stability over time, and finally its ease to weight in comparison to the standard liquid oleate, which is a highly viscous plastic fluid; secondly, the solid oleate presents distinctive characteristics in comparison to the liquid oleate such as a higher Fe content as determined by TG (33 wt% Fe in the solid oleate-Fe against 6 wt% in the liquid iron oleate, Figure 1(c)) and different iron-oleic acid coordination. This means that the reaction using the solid oleate-Fe requires the addition of a larger amount of extra oleic acid to preserve the oleic acid/Fe ratio of 3-4 that has been described as ideal for the synthesis of uniform magnetic nanoparticles by thermal decomposition [16, 23]. The use of a liquid oleate with a composition that changes with time makes the control of the amount of oleic acid in the reaction media difficult, which is critical to control the particle growth and consequently the particle size [23, 24]. Slight differences in composition and oxidation degree of the iron oleates have been further analyzed by gas chromatography coupled with mass spectrometry GC-MS and are included in the supporting information (Figures S1 and Table S1).

On the other hand, solid and liquid oleates have different Fe coordination to the carboxylic groups of the oleic acid as shown by IR spectroscopy (Figures 1(a) and 1(b)), being bidentate in the case of the solid oleate instead of monodentate. This is reflected in the distance between the carboxyl bands at 1600 and 1455 cm^−1^ [25], which is 145 cm^−1^ for the liquid oleate and for the solid oleate is 86 cm^−1^. Solid iron oleate is in fact an iron hydroxide as demonstrated by X-ray diffraction (Figure 1(d)). This oleic coated hydroxide is expected to be less reactive than liquid oleate that present also X-ray diffraction pattern but with low crystallinity (Figure S2). Therefore, the temperature ramp becomes a key parameter to control the solid oleate precursor decomposition and consequently the particle nucleation. Moreover, the solid oleate consists of tiny anisometric nanoparticles (around 10 by 2 nm as shown by TEM Figure S3) that resemble those for Fe hydroxides such as goethite or lepidocrocite [26]. In contrast to that, liquid oleate having monodentate coordination and amorphous structure decomposes easily and is less sensible to the temperature ramp resulting in similar particle sizes for temperature ramps between 3 and 6°C/min [27].

### 3.1. Effect of Key Microwave Synthesis Parameters on Particle Size and Size Distribution

The effect of different key parameters such as solvent, heating ramp, and iron concentration on the microwave assisted synthesis of magnetic nanoparticles has been evaluated. For some selected conditions, the effect of the heating source has also been analyzed in comparison to the thermal heating.

#### 3.1.1. Solvent

When considering solvents for the microwave reaction in a pressurized vessel, boiling points become less important than the efficiency of the reactant mixture to couple with an applied microwave field. Among the different solvents with dielectric constants between 1 (vacuum) and 88 (water), solvents with dielectric constants from above 2 (octadecene) to 47 (DMSO) were chosen. It was observed that too polar solvents with relatively high vapor pressure, like DMSO, generate a fast built up pressure in the system and the equipment shuts down, as a safety precaution. On the other side, solvents with low dielectric constant, such as octadecene, generate paramagnetic nanoparticles (Figure S4). Dibenzyl ether, with a dielectric constant of 3.86, was chosen as the best one in this case, given the magnetic properties of synthesized nanoparticles, and the price that is 10 times lower than benzyl alcohol, solvent utilized by the majorities of research groups using microwave heating [4, 7–9, 30].

#### 3.1.2. Heating Ramps

**Figure 2:**
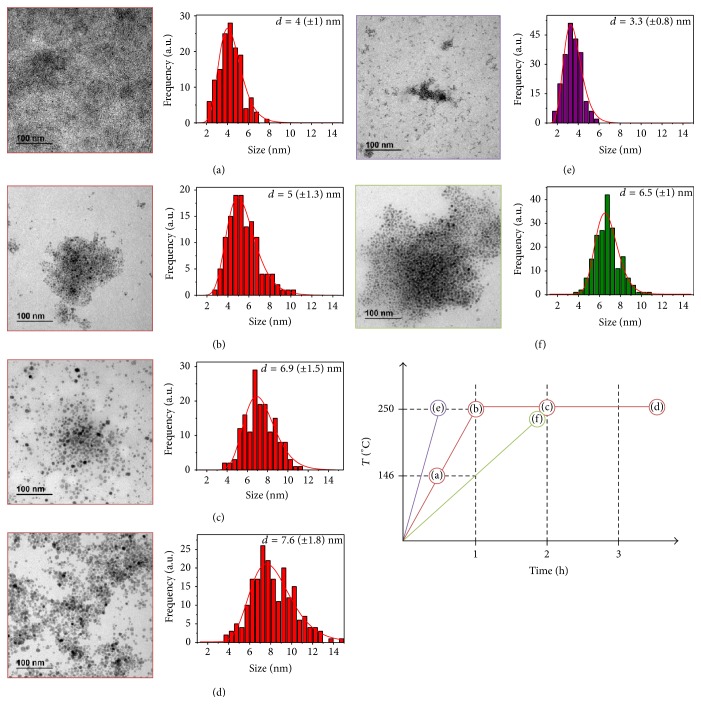
TEM images of the nanoparticles, with its respective size distribution, obtained with different heating ramps and total synthesis time, with a schematic figure of these ramps. (a) 146°C in 30 minutes, (b) 250°C in 1 hour, (c) 250°C in 1 hour and maintained for 1 hour, (d) 250°C in 1 hour and maintained for 2 hours and 30 minutes, (e) 250°C in 30 minutes, and (f) 250°C in 2 hours.

Basically three heating ramps were tested as shown in Figures 2(b), 2(e), and 2(f), that is, 3.75, 7.5, and 1.8°C/min. The optimal ramp is 3.75°C/min that gives rise to uniform nanoparticles of around 5 nm (Figures 2(a) and 2(b)). Faster heating ramps result in smaller nanoparticles, 3.3 nm (Figure 2(e)), while slower heating ramps result in larger particles with heterogeneous geometry, 6.5 nm, main diameter (Figure 2(f)). Prolonging the reaction time from 1 up to 4 hours it is possible to get larger uniform particles up to a limit of around 8 nm, which is given by the exhaustion of iron precursor (Figures 2(c) and 2(d)) [24].

#### 3.1.3. Iron Concentration

Other important parameters to consider in iron oxide nanoparticle synthesis is the iron concentration. Different tests were carried out as presented in Figure S5 and summarized on Table 1. From Figure S5 (A) to (D), four samples with different iron concentration are shown while the heating ramp and the oleic acid/Fe molar ratio were kept constant. It can be observed that the higher the iron concentration (from 2 to 5 mg Fe/ml), the smaller the particles and the size distribution, always below 25%. Similar results were found for thermal decomposition [31]. However, at 4 mg Fe/ml concentration, particle size is not affected by the addition of three times more oleic acid to the reaction mixture (Figure S5, from E to H) (slight reduction in size from 6.5 to 5.7 nm and narrower distribution, 20%) in contrast with the strong oleic acid effect when using other iron precursors such as iron acetylacetonate [32]. In that case, Fe(acac)_3_ decomposes forming an intermediate iron-oleate complex at 200°C and nucleation takes place at higher temperatures as the concentration of oleic acid increases leading to an important reduction in particle size.

#### 3.1.4. Heating Source

**Figure 3:**
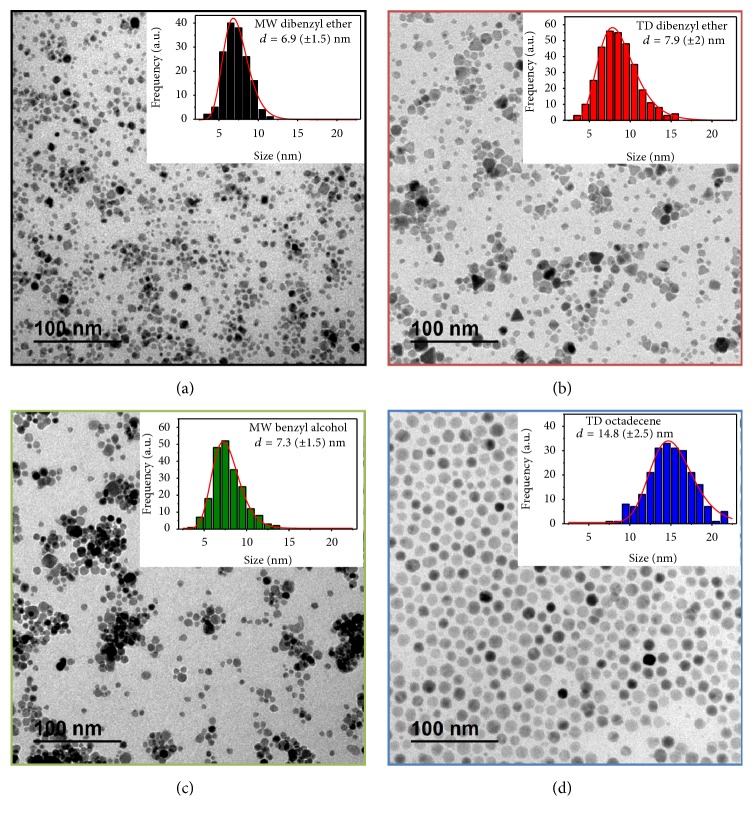
TEM images of magnetite nanoparticles obtained by microwave (MW) and thermal decomposition (TD), using different solvents (dibenzyl ether (a and b), octadecene (d), and benzyl alcohol (c)). Nanoparticles coated with DMSA. Descriptions of the samples are included in Table 2.

Heating method is expected to play an important role on the final size and distribution of the nanoparticles. To analyze this the same reaction mixture was decomposed with microwave and thermal heating. The parameters chosen as default are iron concentration of 4 mg Fe/ml, molar ratio (oleic/Fe) of 5, heating ramp of 3.75°C/min until 250°C, kept for 1 hour at this temperature, and solid iron oleate as iron precursor. Microwave samples were synthesized using dibenzyl ether (Figure 3(a)) and benzyl alcohol (Figure 3(c)) as solvents (MwE8 and MwA8), while the samples prepared by thermal decomposition were obtained using dibenzyl ether (Figure 3(b)) and octadecene (Figure 3(d)) as solvents (TdE12 and TdO15).  Figure 3 shows their respective TEM images, with the same magnification for ease comparison. Insets show their size distribution fitted by Log-normal. Mean size and distribution are included in Table 2. For sample MwE8 the mean size is 6.9 nm (*σ* = 0.21), while MwA8 has a size of 7.3 nm (*σ* = 0.21). On the other side, TdE12 has a mean size of 7.9 nm (*σ* = 0.26) while TdO15 has a size of 14.8 nm (*σ* = 0.17).

**Figure 4:**
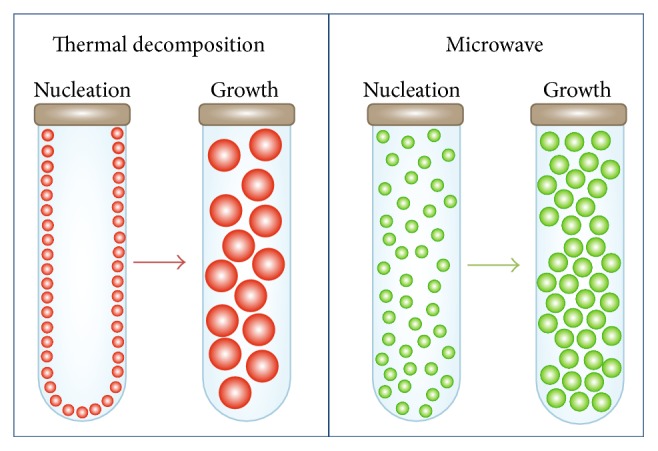
Comparison between thermal decomposition and microwave heating showing the effect on nanoparticles sizes. Nanocrystals tend to nucleate on the vessel wall first for thermal decomposition; on the contrary for microwave nanocrystals tend to form rapidly, creating more seeds that grow less.

First, it should be noted that the largest size is always obtained for the particles prepared in the solvent with the highest boiling point (dibenzyl ether = 160°C, benzyl alcohol = 205°C, and octadecene = 315°C) [14, 24, 33]. In addition, nanoparticles synthesized by heat transfer tend to be larger than the ones produced by microwave heating. This difference in sizes between both synthesis methods can be explained by their different nucleation and growth processes, as seen in Figure 4. Using conventional heating, nanocrystals tend to nucleate on the vessel walls first, given its inhomogeneous heating profile [34]. When a sample is irradiated with microwave frequencies, the dipoles tend to align in the direction of the applied electric field; in such a way energy is lost in the form of heat, through dielectric loss and molecular friction [6]. Given that, microwave produces efficient internal heating, creating numerous “hot spots,” which could trigger multiple nucleation events throughout the solution, increasing the product yield [1, 35] but decreasing the average size due to the enhanced competitive growth.

### 3.2. Effect of the Heating Source on the Structural, Colloidal, and Magnetic Properties of the Nanoparticles

**Figure 5:**
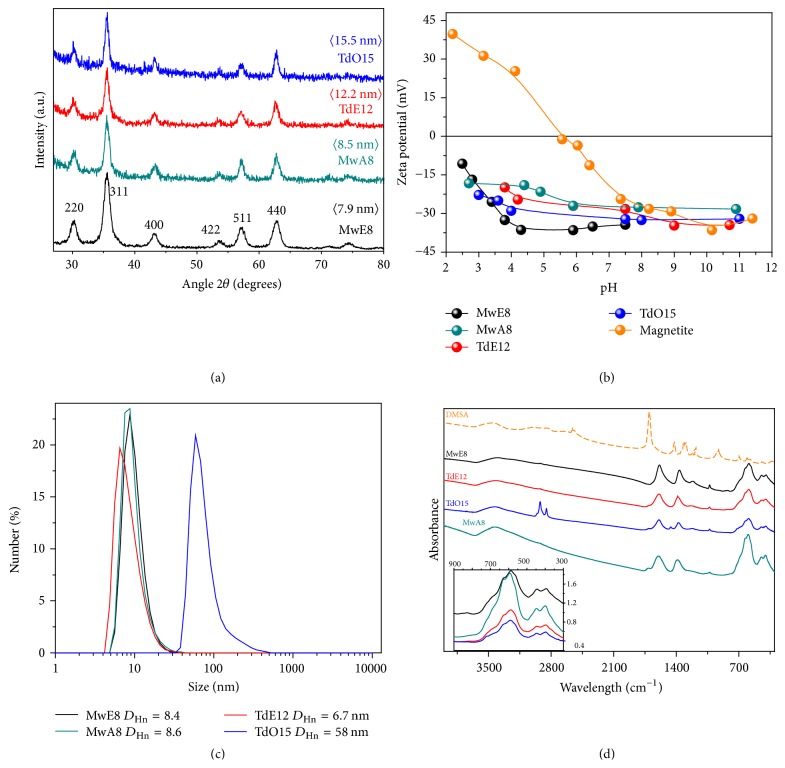
(a) X-ray diffraction patterns with calculated mean size of crystal for magnetite nanoparticles obtained by microwave (MW) and thermal decomposition (TD) using different solvents; (b) zeta potential measurements as a function of pH for DMSA coated nanoparticles and uncoated magnetite for comparison; (c) hydrodynamic size in number distribution; (d) infrared spectra for DMSA coated nanoparticles and DMSA for comparison. Inset shows the IR low frequency range.

Structural characterization of the nanoparticles prepared in this work was carried out by X-ray diffraction.  Figure 5(a) shows the X-ray patterns for nanoparticles obtained by microwave (MW) and thermal decomposition (TD) using different solvents. All peaks correspond to crystallographic magnetite or maghemite planes discarding the presence of secondary phases. Crystal sizes calculated from the broadening of 311 peak vary from 7.9 nm to 8.5 nm for MW samples and from 12.2 nm to 15.5 nm for TD samples. The values differ only slightly from the size distribution obtained by TEM images indicating the single-core character of the particles.

Nanoparticles were transferred to an aqueous medium by exchanging the oleic acid of the surface by dimercaptosuccinic acid (DMSA) [36]. DMSA coating on nanoparticles is responsible for the high negative charge (between −27 and −34 mV) in a wide pH range, between pH 2 and 11 (Figure 5(b)). Hydrodynamic sizes in intensity are between 30 and 170 nm (Figure S6), increasing as the amount of coating on the nanoparticle surface increases, as it can be seen from the thermogravimetric analysis (Figure S7 and S8). For example, sample TdO15, which has the larger hydrodynamic size, has a larger amount of DMSA on the nanoparticle surface, but the presence of aggregates cannot be completely discarded. Hydrodynamic sizes in number are adjusted to a monomodal distribution with mean values around 6–9 nm for the smallest particles and 58 nm for the largest ones, that is, TDO15 (Figure 5(c)). This means that most of the particles are well dispersed forming a stable colloid and only a small fraction are aggregated leading to a broadening of the peak in the DLS intensity distribution or a bimodal distribution, as it is the case of sample MwA8 (Figure S6). No significant differences were found for particles prepared by MW or by TD in relation to the colloidal properties. Hydrodynamic sizes are important depending on the application since it may limit its use. For example, for hydrodynamic sizes larger than 200 nm, particles are rapidly captured by the Reticuloendothelial System (RES) or cell uptake is interfered [36].

Infrared spectra of these nanoparticles show the typical bands for water above 3100 cm^−1^, at 3000 cm^−1^ for C-H vibration, between 1000 and 1700 cm^−1^ for the coating signature and bands below 1000 cm^−1^ associated with the vibration modes of the iron oxide, Fe-O stretching [37] (Figure 5(d)). Infrared spectra of the nanoparticles coated with oleic acid are presented on Figure S9.

**Figure 6:**
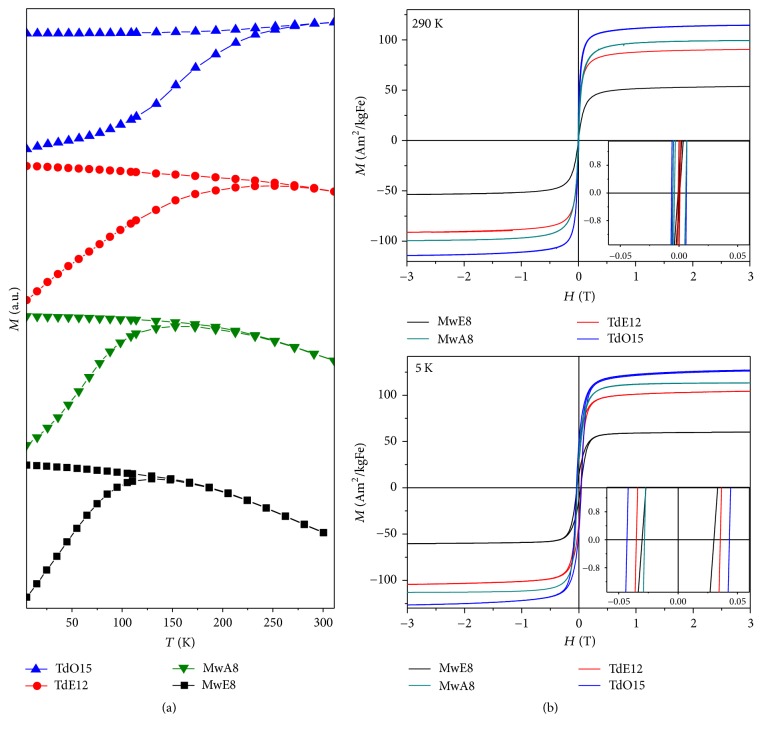
Hysteresis loops at room temperature and 5 K (b) and zero field cooled (ZFC)/field cooled (FC) magnetization curves measured at 100 Oe (a) for DMSA coated magnetite nanoparticles obtained by microwave (MW) and thermal decomposition (TD) using different solvents. The loops were fitted by Langevin function.

Magnetic properties were analyzed for DMSA coated nanoparticles at room temperature and 5 K (Figure 6). The saturation magnetization values and nanoparticle magnetic size were achieved by fitting the magnetization curves at room temperature to the Langevin function taking into account the particle size log normal distribution (Table 2). The hysteresis loops show that the larger the particle, the larger their saturation magnetization due to the decrease in surface area/volume ratio, and therefore the lower the surface effects such as spin canting [16, 38]. The higher saturation magnetization values for TdO15 are close to those reported for bulk magnetite (115–130 emu/gFe at RT and 5 K) [21]. The smallest values (55–60 emu/gFe) correspond to MwE8 sample with the smallest crystal size (Figure 5(a)). However, sample MwA8 with smaller crystal size than TdE12 has higher saturation magnetization suggesting less spin canting due to internal or surface disorder for the sample prepared by the microwave assisted route. At room temperature these nanoparticles are close to the superparamagnetic regime, showing rather low coercive fields, while at 5 K the systems are magnetically blocked, showing higher coercivity for larger particles [38, 39]. The initial susceptibility values increase as the particle size increases, given that the number of magnetic moments that align with the field grows. Magnetic particles sizes calculated from the Langevin function vary from 6.4 nm (*σ* = 0.33) for MwE8 up to 8.8 nm for TdO15 (*σ* = 0.27) (Table 2). The differences between TEM and magnetic size for TD samples suggest a strong influence of magnetic interactions on the *M*/*H* curves for these samples with larger particle sizes.

Figure 6 shows the ZFC-FC curve for all samples; they are measured from 20 K to room temperature. Where the blocking at low temperature is evident for smaller nanoparticles (MW), bigger particles are still blocked at room temperature (TD) [16].

**Figure 7:**
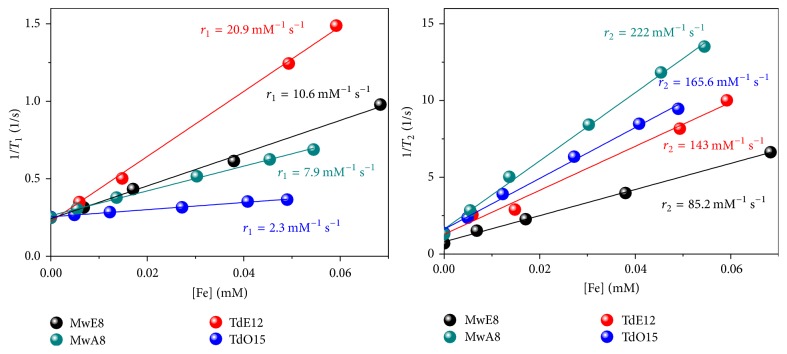
MRI *T*_1_ and *T*_2_ relaxation times as a function of the Fe concentration for DMSA coated magnetite nanoparticle suspensions obtained by microwave (MW) and thermal decomposition (TD) using different solvents.

Measurements of the MRI relaxation times (*T*_1_ and *T*_2_) were made at different iron concentrations from 0 to 0.07 mM Fe to obtain the relaxivity value (*r*_1_ and *r*_2_), as seen in Figure 7. The maximum *r*_1_ value (20.9 mM^−1^ s^−1^) was found for a TD sample, while the maximum *r*_2_ value (222 mM^−1^ s^−1^) was found for a MW sample. Samples obtained by thermal decomposition with the largest particle size (TdO15) present extremely low *r*_1_, and consequently much higher *r*_2_/*r*_1_ ratio probably as a consequence of the larger hydrodynamic size (Figure 5(c)). On the other hand, samples synthesized by microwave assisted route have similar *r*_1_ values but sample MwA8 presents more than double *r*_2_ value, indicating higher efficiency as negative contrast agent. In the literature, the maximum experimental value reported for *r*_2_ is around 500 mM^−1^ s^−1^ for iron oxide nanoparticles, while the theoretical maximum value is 750 mM^−1^ s^−1^, not yet reached [15]. Commercial formulations using magnetic nanoparticles used for pathology diagnosis in the liver and spleen, as Feridex®, produced by Berlex Laboratories and Resovist, produced by Bayer Healthcare have *r*_1_ and *r*_2_ values between 24 and 150 mM^−1^ s^−1^ and *r*_2_/*r*_1_ of around 4–6 [40]. Looking at the relation between *r*_2_ and *r*_1_ for our samples it can be seen that samples MwA8 and TdO15 have high possibility to become *T*_2_ contrast agents, with a quotient of 28.1 and 72, respectively.

The relaxivity at high magnetic fields for the particles synthesized in this work is expected to follow the motional average regime (MAR) that describes the interaction of the nanoparticles with water protons taking into account the nanoparticle size (TEM) and the magnetic field distribution around it [41]. Therefore, the relaxivity *r*_2_ is given by

(1) $$\begin{array}{c} r_{2}=\frac{4\gamma^{2}\mu_{0}^{2}\nu_{\text{mat}}M_{v}^{2}d^{2}}{405D}, \end{array}$$

where *γ* = 2.67513 × 10^8^ rad·s^−1^·T^−1^ is the gyromagnetic factor of the proton, *μ*_0_ = 4*π*10^−7^ T·m·A^−1^ is the magnetic permeability of vacuum, *D* is the water translational diffusion constant, *d* is the particle diameter, *ν*_mat_ is the molar volume of the material (1.5 × 10^−5^ m^3^/mol for magnetite), and *M*_*v*_ is the saturation magnetization expressed in SI units, A·m^−1^ [15, 41].

**Figure 8:**
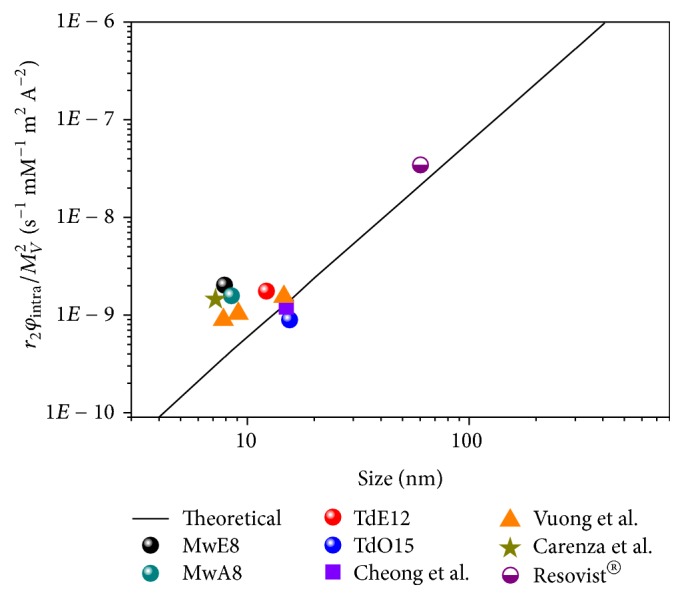
Influence of the core size calculated by TEM on *r*_2_ relaxivity normalized by the square of the saturation magnetization. Colorful dots correspond to the nanoparticles studied on this work and compared to others works (orange triangles [15], purple square [29], and star [4]) and compared to commercial one Resovist® (purple circle); the solid line corresponds to the theoretical values for the motional averaging regime (MAR). For the calculations *ψ*_intra_ = 1 was used for all samples but the Resovist, which used *ψ*_intra_ = 8.4*E* − 2.

Figure 8 shows theoretical (straight line) and experimental (symbols) *r*_2_ values for magnetic nanoparticles of different sizes (iron oxide core measured by TEM) normalized by the square of the saturation magnetization [15]. Experimental values obtained in this work together with other reported data for particles of similar core size prepared by microwave synthesis (Figure 8 star [4]), thermal decomposition (Figure 8 purple square [29]), Massart's procedure [42] (Figure 8 orange triangles [15]), or the commercial one Resovist (Figure 8, purple circle) are included. Deviation from the theoretical curve may be due to differences in intra-aggregate volume fraction, that is, the number of cores per aggregate. In the case of the nanoparticles obtained in this work *ψ*_intra_ = 1 was used given the single-core character supported by TEM and DLS measurements. It should be noted that *r*_2_/*M*_*v*_^2^ is almost constant with particle size for all samples studied in this work. Saturation magnetization seems to be the main parameter controlling the efficiency of these nanoparticles as MRI *T*_2_-contrast agents independently of the particle size.

## 4. Conclusions

The microwave assisted synthesis has been explored for the preparation of magnetic iron oxide nanoparticles showing the critical effect of different experimental parameters such as the solvent, the precursor, and the surfactant on the nucleation and growth processes that determine particle size and uniformity. Dibenzyl ether was chosen as the best solvent for this synthesis given its dielectric constant, the optimal ramp was set at 3.75°C/min, the iron concentration was 4 mgFe/ml, and (oleic/Fe) molar ratio was 5 giving rise to the most uniform nanoparticles. In comparison to conventional heating where nanocrystals tend to nucleate on the vessel walls first given its inhomogeneous heating profile, microwave produces efficient internal heating promoting nucleation everywhere and reducing the growth possibilities of the numerous nuclei generated.

Magnetic iron oxide nanoparticles with sizes between 8 and 15 nm synthesized by microwave and thermal decomposition in organic media present nearly superparamagnetic behavior at RT and relaxivity values that make them good candidates for MRI negative contrast agents. Given the low hydrodynamic size of these suspensions, saturation magnetization seems to be the main parameter controlling the efficiency of these magnetic nanoparticles as MRI *T*_2_-contrast agents.

## Figures and Tables

**Table 1:** Comparison of nanoparticle sizes when changing the iron concentration and the oleic acid/Fe molar ratio.

Molar ratio 5 (Oleic/Fe)	[Fe] mg/ml	2	3	4	5
	*d* (*σ*) nm	8.2 (±2.2)	9.7 (±2.7)	6.9 (±1.5)	6.7 (±1.7)

[Fe] = 4 mg/ml	Molar ratio (oleic/Fe)	2	3.5	5	6.5
	*d* (*σ*) nm	6.5 (±1.3)	7.1 (±2.2)	6.9 (±1.5)	5.7 (±1.2)

**Table 2:** Comparison of structural and magnetic properties^a^ for all samples described on the manuscript.

	MwE8	MwA8	TdE12	TdO15
Diameter TEM (nm) number	6.9 (0.21)	7.3 (0.21)	7.9 (0.26)	14.8 (0.17)
Diameter TEM (nm) volume	8.1	9	10.5	16.3
Diameter XRD (nm)	7.9	8.5	12.2	15.5
Diameter DLS (nm)	23 (0.2)	68 (0.5)	37 (0.3)	173 (0.3)
Diameter VSM (nm)	6.4 (0.33)	7.1 (0.4)	8.5 (0.29)	8.8 (0.27)
Volume XRD (10^3^ nm^3^)	2.1	2.5	7.6	15.6
*M*_*S*_ at 5 K (Am^2^/kgFe)	60	114	105	128
*M*_*S*_ at RT (Am^2^/kgFe)	55	100	93	115
*H*_*C*_ at 5 K (10^4^ A/m)	2.4	2.2	2.8	3.2
*H*_*C*_ at RT (10^4^ A/m)	0.47	0.21	0.18	0.63
Zeta potential (mV) at pH 7	−34.4	−27.6	−28.3	−32.2
*r*_2_/*r*_1_	8	28.1	6.8	72
*r*_2_ (mM^−1^ s^−1^)	85.2	222	143	165.6
*r*_1_ (mM^−1^ s^−1^)	10.6	7.9	20.9	2.3

^a^Diameter DLS is *Z*_average_ and the number between brackets is the polydispersity index; *M*_*S*_ = saturation magnetization; *H*_*C*_ = coercive field; *r*_2_ = MRI transversal relaxivity; *r*_1_ = MRI longitudinal relaxivity.

## Supplementary Materials

Supplementary Materials Figure S1. GC-MS trace (total ion chromatogram) of the methylated fatty acid composition of the solid iron oleate (A) and liquid iron oleate (B). Table S1. Fatty acid composition of synthesized iron oleates. Figure S2. X-ray diffraction pattern for liquid iron oleate. Figure S3. TEM image of solid iron oleate, where a hydrophobized hydroxide nanomaterial can be seen. Figure S4. Hysteresis loops at room temperature for nanoparticles obtained by microwave using different solvents. Solvents with low dielectric constant such as octadecene and phenyl ether generate paramagnetic nanoparticles while solvents with higher dielectric constant generate nanoparticles with high saturation magnetization values. Figure S5. TEM images and size distribution of the nanoparticles obtained when changing the iron concentration from 2 to 5 mg Fe/ml (35.8 mM to 89.5 mM), shown on images A to D. On the other hand, changing the molar ratio oleic acid/Fe (MR) from 2 to 6.5, images E to H. Red lines represent a Log-normal fit. Figure S6. Hydrodynamic size in intensity distribution is between 30 and 170 nm for all samples. Figure S7. Thermogravimetric analysis showing the amount of DMSA coating on nanoparticles obtained by microwave (MW) and thermal decomposition (TD), using different solvents. DMSA weight loss is included for comparison. Figure S8. Thermogravimetric analysis showing the amount of oleic acid coating on nanoparticles obtained by microwave (MW) and thermal decomposition (TD) using different solvents. Figure S9. Infrared spectra for oleic acid coated nanoparticles obtained by microwave (MW) and thermal decomposition (TD) using different solvents. The inset shows the IR low frequency range.
