# Supplementary material for: Key Parameters on the Microwave Assisted Synthesis of Magnetic Nanoparticles for MRI Contrast Agents
Source: Contrast Media Mol Imaging. 2017 Dec 4;2017:8902424. doi: 10.1155/2017/8902424 (PMC5733996; doi:10.1155/2017/8902424)
Supplement: Supplementary Materials — Figure S1. GC-MS trace (total ion chromatogram) of the methylated fatty acid composition of the solid iron oleate (A) and liquid iron oleate (B). Table S1. Fatty acid composition of synthesized iron oleates. Figure S2. X-ray diffraction pattern for liquid iron oleate. Figure S3. TEM image of solid iron oleate, where a hydrophobized hydroxide nanomaterial can be seen. Figure S4. Hysteresis loops at room temperature for nanoparticles obtained by microwave using different solvents. Solvents with low dielectric constant such as octadecene and phenyl ether generate paramagnetic nanoparticles while solvents with higher dielectric constant generate nanoparticles with high saturation magnetization values. Figure S5. TEM images and size distribution of the nanoparticles obtained when changing the iron concentration from 2 to 5 mg Fe/ml (35.8 mM to 89.5 mM), shown on images A to D. On the other hand, changing the molar ratio oleic acid/Fe (MR) from 2 to 6.5, images E to H. Red lines represent a Log-normal fit. Figure S6. Hydrodynamic size in intensity distribution is between 30 and 170 nm for all samples. Figure S7. Thermogravimetric analysis showing the amount of DMSA coating on nanoparticles obtained by microwave (MW) and thermal decomposition (TD), using different solvents. DMSA weight loss is included for comparison. Figure S8. Thermogravimetric analysis showing the amount of oleic acid coating on nanoparticles obtained by microwave (MW) and thermal decomposition (TD) using different solvents. Figure S9. Infrared spectra for oleic acid coated nanoparticles obtained by microwave (MW) and thermal decomposition (TD) using different solvents. The inset shows the IR low frequency range. [file 8902424.f1.pdf]

Supporting Information

Key Parameters on the Microwave Assisted Synthesis of Magnetic Nanoparticles.

Maria Eugênia Fortes Brollo [1], Sabino Veintemillas-Verdaguer [1], Cesar Menor Salván [2,3], Maria del Puerto Morales [1]

[1] *Institute of Material Science of Madrid, ICMM-CSIC, Sor Juana Inés de la Cruz 3, 28049 Madrid, Spain.*
[2] *School of Chemistry and Biochemistry, Georgia Institute of Technology. 315 Ferst Drive NW, Atlanta, GA 30332 USA*
[3] *IMIDRA, Finca El Encin, Autovía del Noreste A-2, Km. 38.200, 28805 Alcalá de Henares, Madrid, Spain.*

CHARACTERISATION OF LIQUID AND SOLID IRON OLEATES

EXPERIMENTAL PROCEDURES

To determine the organic composition, the synthesized iron oleate was analyzed by gas chromatography coupled with mass spectrometry (GC-MS). Before analysis, the fatty acid salts were converted in its corresponding fatty acid methyl esters (FAMEs) by treatment with methanol/hydrochloric acid solution, followed by extraction of FAMEs in hexane. A mixture of 1 ml methanol and 3 ml of HCl 3M in methanol was added to 50 mg of oleate sample in a 50 ml rounded flask. The mixture was refluxed for 15 minutes. After cooling, 8 ml of NaCl 0.8% were added and the mixture was extracted by vigorous stirring with 3 ml of n-hexane. The organic fraction was collected and the aqueous fraction, containing iron (III) chloride complexes was discarded. During the process, no interfering quantities of $FeCl_3$ were extracted together with the organic fraction. As an alternative procedure, to test potential interfering iron in the organic extract, we added 8 ml of NaOH 0.5 M solution after cooling, to precipitate the iron oxy-hydroxides. We did not observe significant differences in the result and excess of NaOH could result in the saponification of FAMEs. In subsequent analyses we followed the first standardized procedure.

The hexane extract was analyzed without further treatment in an Agilent 6850 GC coupled to an Agilent 5975 C single quadrupole mass spectrometer. Separation was achieved on a OV-5MS (Ohio Valley Specialty) column coated with 5% diphenyl, 95% dimethylpolysiloxane (30 m x 0.25 mm, 0.25 µm film thickness) and on a DB-17 column coated with (50%-phenyl)-methylpolysiloxane. The operating conditions were as follows: 8 psi He carrier gas pressure, initial temperature hold at 40 ºC for 1.5 min, increased from 40 to 150 ºC at a rate of 15 ºC/min, held for 2 min, increased from 150 to 255 ºC at a rate of 5 ºC/min, held isothermal for 20 min, and finally increased to 300 ºC at a rate of 5 ºC/min. The sample was injected in the splitless mode with the injector temperature at 290 ºC. The mass spectrometer was operated in the electron impact mode at 70 eV ionization energy and scanned from 40 to 700 Da. The temperature of the ion source was 230 ºC and the quadrupole temperature was 150 ºC. Data were acquired and processed using the Agilent Chemstation software, and percentages calculated by normalization of peak areas of the corresponding compounds in the total extracts. Identification of FAMEs was based on mass spectra interpretation and comparison of spectra and retention times with the Certified Reference Material Supelco 37 component FAME mix (CRM47885, Supelco, USA).

RESULTS

The analysis shows that the starting oleic acid used in the preparation of the solid iron oleate and the sodium oleate used for the preparation of liquid iron oleates were not totally pure, containing significant quantities of palmitic, palmitoleic, myristic, stearic and vaccenic acids. This composition and the presence of the homologous series of cis-omega 9 monounsaturated fatty acids, evidences a natural origin for the oleic acid and sodium oleates used in the synthesis of iron oleates. The solid oleate presents less proportion of secondary fatty acids other than oleic acid Figure 1 Table 1. It is noticeable the formation of oxidation product 9,10-epoxystearic acid and small quantities of azelaic acid and nonanoic acid, formed by oxidative cleavage of oleic acid. These oxidation products are present at significant concentration, suggesting that rather the iron oleate synthetic procedure favors the oleic acid oxidation or rancidity of the oleate reagent. The oxidation products are found in higher concentration in the solid iron oleate. This result could be explained by the higher proportion of unsaturated fatty acids, more prone to oxidative degradation.

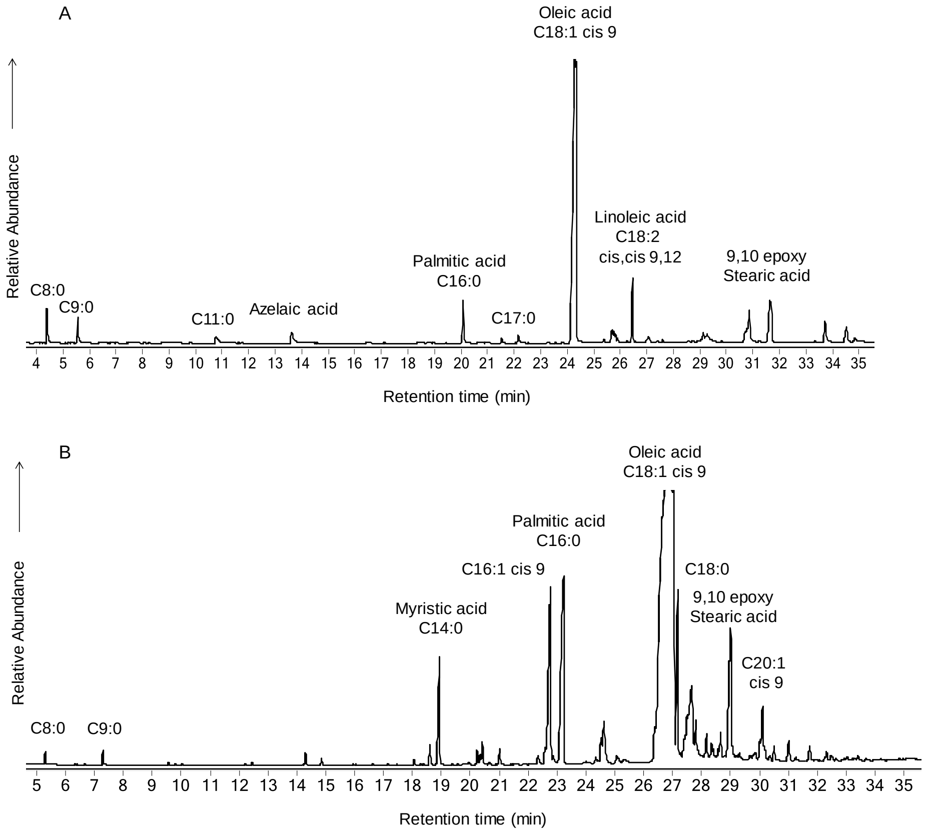

Figure S1. GC-MS trace (total ion chromatogram) of the methylated fatty acid composition of the solid iron oleate (A) and liquid iron oleate (B).

Table S1. Fatty acid composition of synthesized iron oleates

Fatty acid		Liquid Iron Oleate[a,b]	Solid iron Oleate[a,c]
Common name	Lipid notation		
Oleic acid	C18:1 cis 9	62.0	74.11
Stearic acid	C18:0	4.5	-
Linoleic acid	C18:2 cis 9,11	1.0	6.24
Vaccenic acid	C18:1 cis 11	4.1	-
Palmitic acid	C16:0	8.8	3.9
Palmitoleic acid	C16:1 cis 9	6.5	-
Myristic acid	C14:0	3.0	-
Margaroleic acid	C17:1 cis 9	1.8	-
Margaric acid	C17:0	1.5	0.9
Gadoleic acid	C20:1 cis 9	1.9	-
Caprylic acid	C8:0	0.9	2.6
Azelaic acid		0.5	2.5
Oxidation products		5.8	9.8

[a] expressed as % of total organic components; [b] Fig 1B; [c] Fig 1A

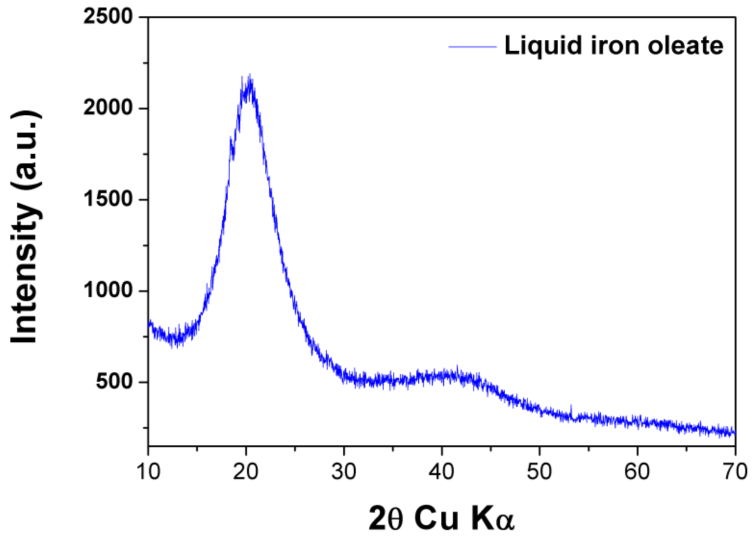

Figure S2: X-ray diffraction pattern for liquid iron oleate.

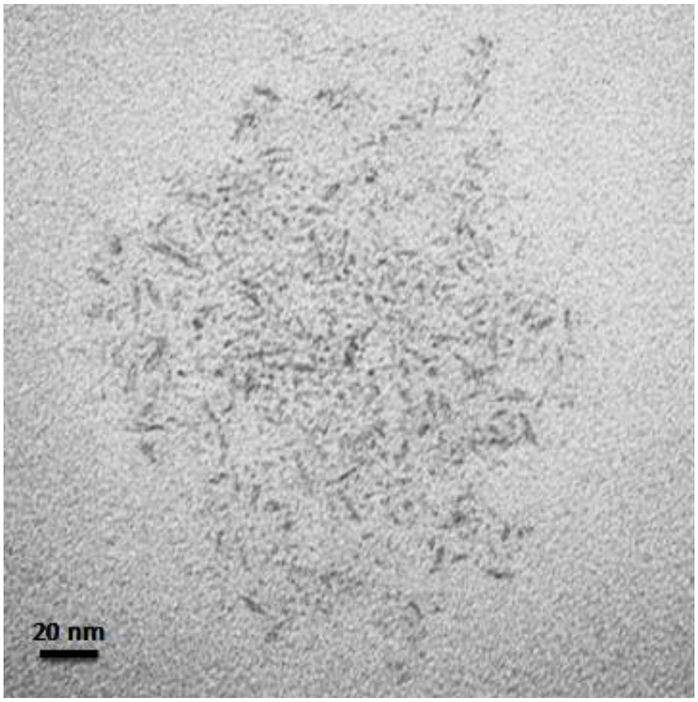

Figure S3: TEM image of solid iron oleate, where it can be seen a hydrophobized hydroxide nanomaterial.

EFFECTS SOLVENT

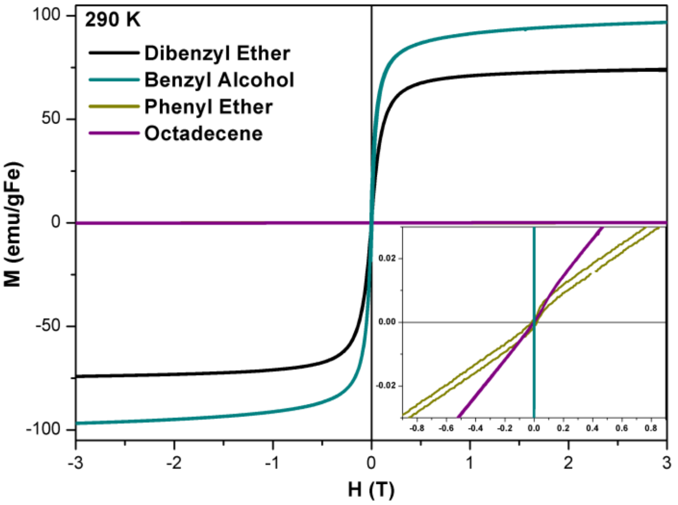

Figure S4: Hysteresis loops at room temperature for nanoparticles obtained by microwave using different solvents. Solvents with low dielectric constant such as octadecene and phenyl ether generate paramagnetic nanoparticles while solvents with higher dielectric constant generate nanoparticles with high saturation magnetization values.

EFFECT OF THE IRON CONCENTRATION

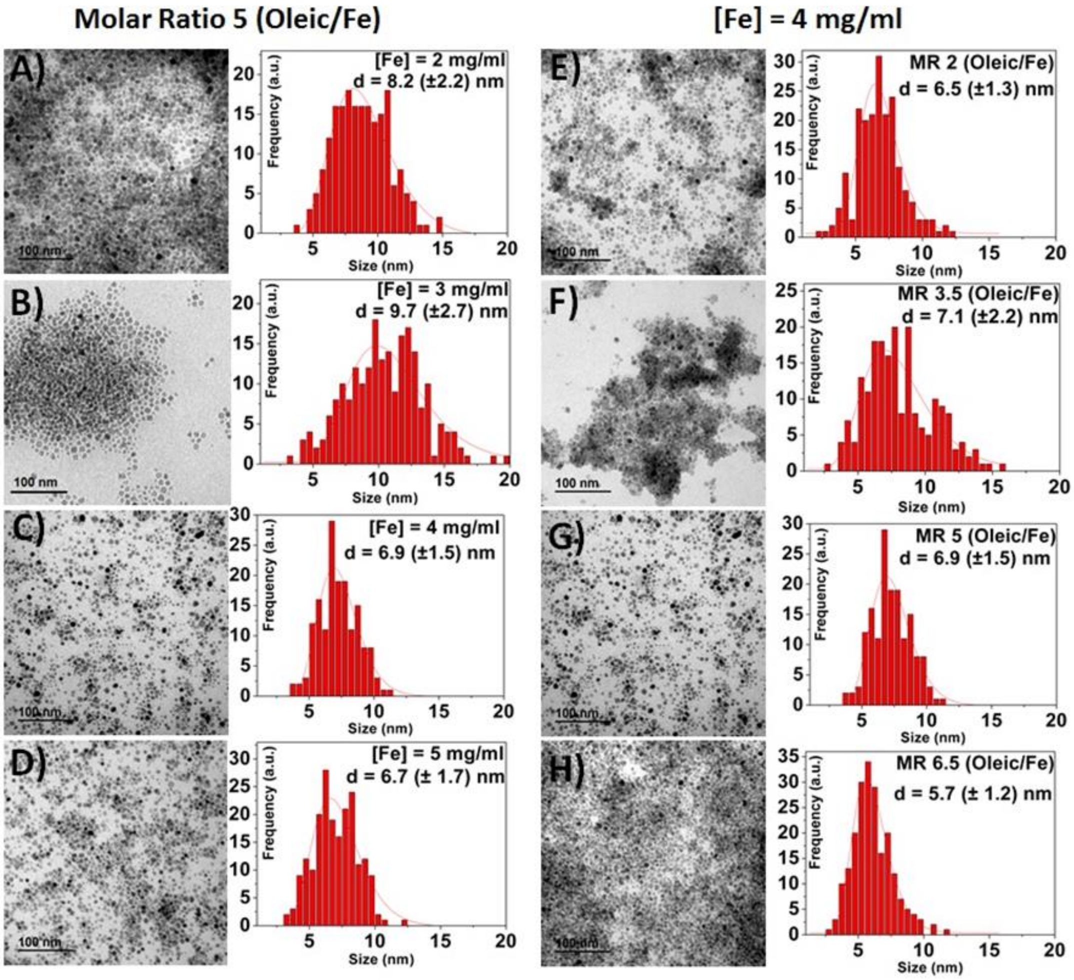

Figure S5: TEM images and size distribution of the nanoparticles obtained when changing the iron concentration from 2 to 5 mg Fe/ml (35.8 mM to 89.5 mM), shown on images A to D. On the other hand, changing the molar ratio Oleic acid/Fe (MR) from 2 to 6.5, images E to H. Red lines represent a Log-normal fit.

EFFECT OF THE HEATING SOURCE

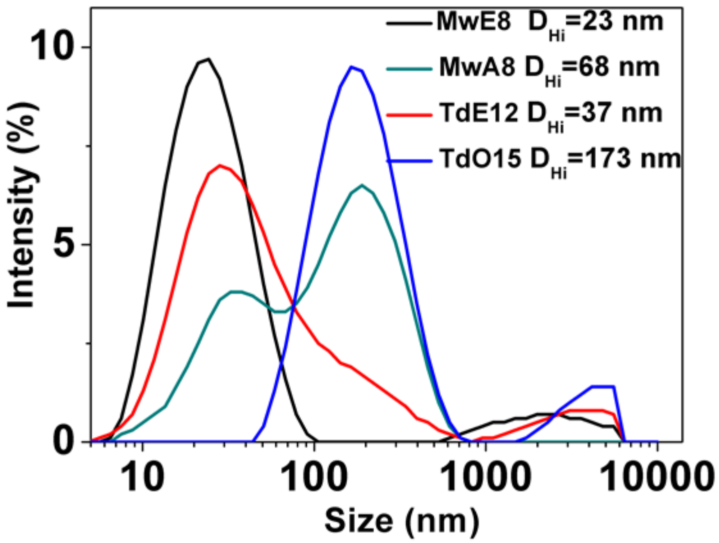

Figure S6: Hydrodynamic size in intensity distribution are between 30 and 170 nm for all samples.

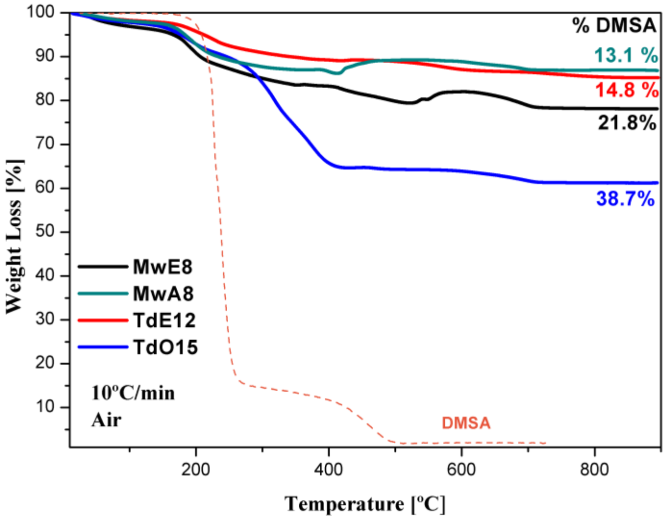

Figure S7: Thermogravimetric analysis showing the amount of DMSA coating on nanoparticles obtained by microwave (MW) and thermal decomposition (TD), using different solvents. DMSA weight loss is included for comparison.

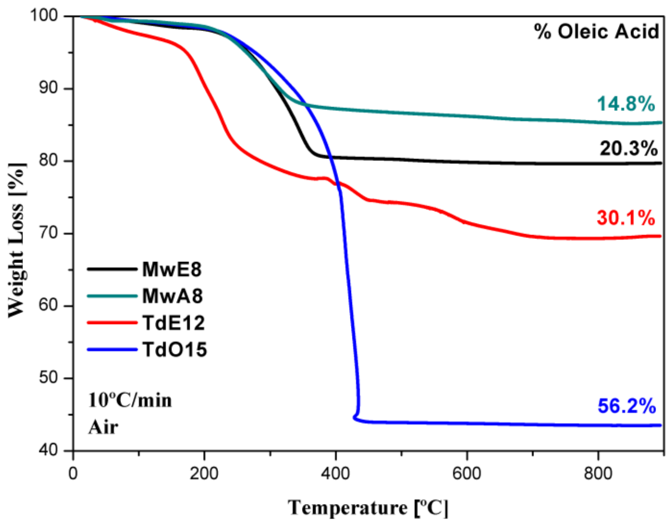

Figure S8: Thermogravimetric analysis showing the amount of oleic acid coating on nanoparticles obtained by microwave (MW) and thermal decomposition (TD) using different solvents.

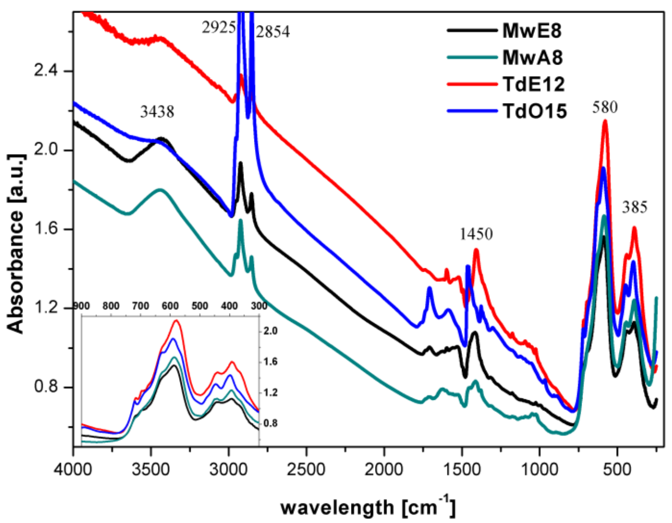

Figure S9: Infrared spectra for oleic acid coated nanoparticles obtained by microwave (MW) and thermal decomposition (TD) using different solvents. The inset shows the IR low frequency range.
